# Perinatal depression and anxiety symptoms as mediators between grief and PTSD: the moderated effect of social support

**DOI:** 10.1186/s12991-025-00603-8

**Published:** 2025-10-10

**Authors:** Jing Zeng, Sheng-Bin Guo, Qing-Xiang Zheng, Zhu-Mei Lin, Xiu-Wu Liu

**Affiliations:** 1 Fujian Maternity and Child Health Hospital, College of Clinical Medicine for Obstetrics & Gynecology and Pediatrics, Fujian Medical University, Fuzhou City, Fujian Province China; 2 Fujian Obstetrics and Gynecology Hospital, College of Clinical Medicine for Obstetrics & Gynecology and Pediatrics, Fujian Medical University, Fuzhou City, Fujian Province China

**Keywords:** Perinatal loss, Perinatal grief, Perinatal depression and anxiety, Social support, Posttraumatic stress symptoms, A moderated mediation model

## Abstract

**Background:**

Posttraumatic stress symptoms are prevalent mental phenomenon in women with previous perinatal loss due to high grief, high perinatal depression and anxiety or low social support. Although posttraumatic stress symptoms are known to have serious negative implications for women with previous perinatal loss, families and society, the mechanism through which it functions is less clear.

**Objective:**

The aim of this study was to examine the moderated mediating effect of social support on perinatal anxiety and depression and its associations with grief and posttraumatic stress symptoms in women with previous perinatal loss. We hypothesized that perinatal depression and anxiety would mediate relationships between grief and posttraumatic stress symptoms and that its mediating effects would differ depending on social support.

**Methods:**

This study was a multicentre cross-sectional survey conducted from December 2021 to October 2022, involving 346 women during hospitalization for perinatal loss as participants from two public hospitals in China. Self-reported scales were used to measure the level of perinatal depression and anxiety, grief, posttraumatic stress symptoms and social support. The Pearson’s correlation analysis, the PROCESS Macro Model 4 and Model 14 on SPSS were used to analyse the available data.

**Results:**

The positive effect of perinatal grief on posttraumatic stress symptoms was found to be mediated by perinatal depression and anxiety, and this mediating effect was moderated according to social support: the more social support, the weaker the mediating effect of perinatal depression and anxiety was between perinatal grief and posttraumatic stress symptoms. The positive effect of perinatal depression and anxiety on posttraumatic stress symptoms was lowest in the high social support group.

**Conclusions:**

Healthcare providers should closely monitor the psychological well-being of pregnant individuals and implement targeted interventions-such as antenatal education course, group-based prenatal care models, and mindfulness-based therapies (e.g., cognitive behaviour therapy) -to mitigate perinatal anxiety and depression. These measures may also significantly reduce post-traumatic stress symptoms in women with previous perinatal loss and high perinatal grief, particularly among those with insufficient social support.

**Supplementary Information:**

The online version contains supplementary material available at 10.1186/s12991-025-00603-8.

## Highlights

1. We explored associations between perinatal grief, perinatal depression and anxiety and posttraumatic stress symptoms among women with previous perinatal loss.
2. Perinatal depression and anxiety mediated the association between perinatal grief and posttraumatic stress symptoms.
3. Low social support exacerbated the effect of perinatal depression and anxiety on posttraumatic stress symptoms.

**Supplementary Information:**

The online version contains supplementary material available at 10.1186/s12991-025-00603-8.

## Introduction

### Perinatal loss-associated PTSD: epidemiological patterns, impacts, and research gaps

Perinatal loss is one of the most common outcomes of reproductive challenges, with estimates suggesting it affects up to 25% of pregnancies [1, 2]. Women with previous perinatal loss, who experienced the loss of a baby or foetus, often suffer a traumatic experience with serious psychological consequences like posttraumatic stress symptoms or even post-traumatic stress disorder (PTSD) [1, 3]. In addition, traditional Chinese culture places strong emphasis on familial continuity and filial piety, leading to perceptions of perinatal loss as a disruption to familial lineage. Even in rural or conservative regions, stigmatization persists, exacerbating emotional distress among women with previous perinatal loss and significantly compromising their psychological well-being [4]. Empirical findings indicate that the prevalence of post-traumatic stress disorder among women with previous perinatal loss ranges from 21.7% to 49.1% [5–7]. In fact, posttraumatic stress symptoms were known to have serious negative implications for women, offspring, families and society [8–10]. Specifically, these symptoms have been linked to detrimental outcomes such as impaired marital relationships and compromised reproductive decisions [8]. Meanwhile, posttraumatic stress symptoms were associated with chronic physical conditions, such as back and neck pain, headaches, arthritis, heart disease, and stroke [10, 11]. Moreover, there was an elevated risk of mental illness, cardiovascular disease, and type 2 diabetes in offspring affected by posttraumatic stress symptoms [9]. Existing PTSD treatments for women have not been specifically tailored for those with previous perinatal loss. Consequently, further investigation into the mechanisms underlying the generation and development of posttraumatic stress symptoms among women with previous perinatal loss is warranted. Such research would provide evidence-based guidance for developing trauma-focused interventions while also enriching the conceptual framework of reproductive trauma psychopathology through mechanistic insights.

### The interrelationship between perinatal grief, perinatal depression and anxiety, social support and posttraumatic stress symptom

Perinatal grief is a common emotional reaction experienced by women following perinatal loss [12]. Perinatal grief may lead to a range of physical, psychosocial, emotional, and cognitive changes [13]. Women who experience higher levels of perinatal grief are more likely to report feelings of unfairness, guilt, and loss of control [14]. These intense emotions can disrupt an individual’s perception of the world and their fundamental beliefs, thereby perpetuating the trauma and contributing to the development of posttraumatic stress symptoms [15]. Additionally, Heazell ’s study argued that women who experience higher levels of perinatal grief may report more intrusive thoughts, images, and nightmares related to the loss [16]. Therefore, high levels of perinatal grief are strongly associated with posttraumatic stress symptoms [2]. Perinatal grief and posttraumatic stress symptoms share similarities (e.g., both are triggered by a stressful life event and are thought to result from a failure of memory integration) [17]. However, there are also clear clinical differences, such as the range of emotions prompted by the disorder (i.e., fear, shame for posttraumatic stress symptoms and yearning for perinatal grief; with guilt, sadness, and anger common in both) [18]. As such, how perinatal grief affects posttraumatic stress symptoms remains unclear, and more research is needed to clarify factors affecting the relationship.

Some researchers suggest that posttraumatic stress symptoms after perinatal loss may be the result of multiple factors [19]. According to the Diathesis-Stress Model of Posttraumatic Stress Disorder, posttraumatic stress symptoms are not only affected by traumatic events such as grief after perinatal loss, but also by ecological and biological factors [20]. Biological factors encompass genetic composition, inherited traits, and those associated with chronic or prolonged exposure to stress [20]. For instance, pregnant women are particularly prone to emotional states, perinatal anxiety, and depression due to chronic exposure to the stress of pregnancy, including hormonal fluctuations, alterations in body shape, role transitions, and concerns regarding foetal health [21]. Meanwhile, some studies have found that the indirect effect of grief after perinatal loss on posttraumatic stress symptoms may be influenced by an individual’s perinatal emotional state, such as depression and anxiety [22]. Other research has found that perinatal depression and anxiety negatively impact postpartum psychological health, including postpartum depression and posttraumatic stress symptoms [23, 24]. Additionally, perinatal depression and anxiety were found to be positively associated with perinatal grief [25]. Based on this, the present study hypothesizes that perinatal grief may directly affect posttraumatic stress symptoms after perinatal loss, and perinatal depression and anxiety act as mediating variables in the above relationship.

Social support refers to the assistance that individuals receive from their social networks, organizational relationships, and the subjective feelings of being understood, supported, and respected by others [26]. According to stress theory, social support is believed to have a buffering effect, and individuals with poor social support from family and friends are more likely to experience posttraumatic stress symptoms when they encounter traumatic events [27, 28]. Meanwhile, the study found that cognitive behavioural factors, such as social disconnection, correlate with bereavement-related mental health problems such as prolonged grief and posttraumatic stress symptoms [28]. Therefore, social support plays a significant role in adjusting physical and psychological health after a traumatic event. Pregnant women are prone to perinatal depression and anxiety in the absence of partner support and in poor marital relationships, which can even affect postpartum psychological conditions, such as posttraumatic stress symptoms [29]. High levels of perceived social support are associated with lower levels of posttraumatic stress symptoms among women who have experienced perinatal loss [30]. Furthermore, social support serves as a crucial moderating variable in relation to various factors that influence the adverse impact of individual psychological responses [31]. Therefore, this study posits that social support may play a moderating role in the relationship among perinatal grief, posttraumatic stress symptoms, and perinatal depression and anxiety.

### Study aims and novel contributions

**Fig. 1:**
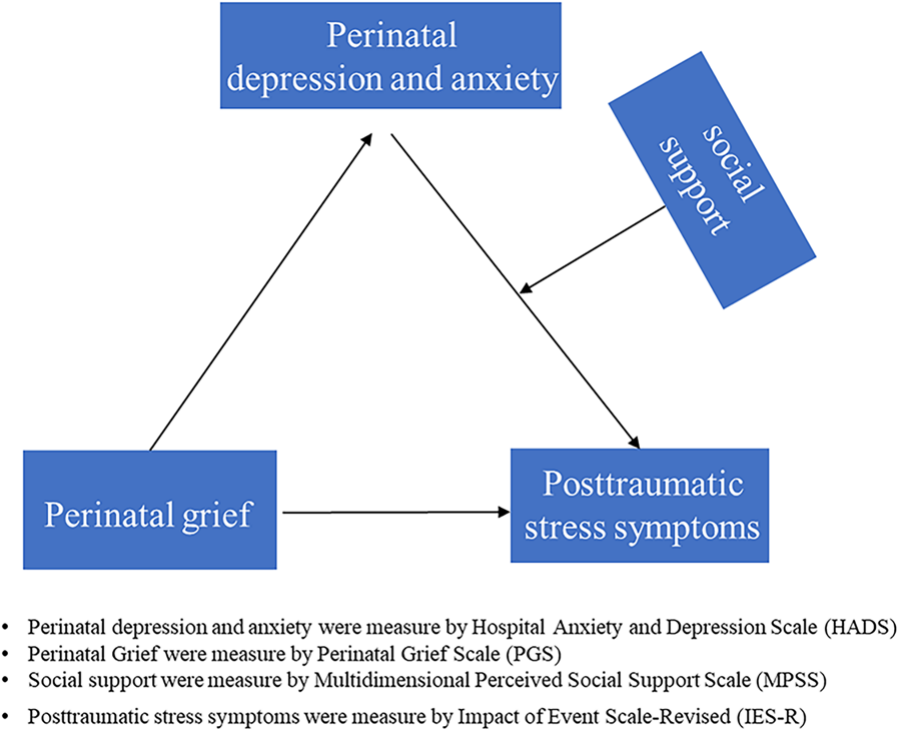
Study conceptual mode

Previous studies have found the individual effects of perinatal grief, perinatal depression and anxiety, and social support on posttraumatic stress symptoms [32–34] However, few studies have analysed these variables in the same model and then examined their interacting effects on posttraumatic stress symptoms among women who have experienced perinatal loss. Therefore, the primary aim of this study was to explore the interrelationships among perinatal grief, posttraumatic stress symptoms, perinatal depression and anxiety, and social support among women who have experienced perinatal loss, according to the Diathesis-Stress Model of Posttraumatic Stress Disorder and Stress Theory. In particular, we sought to determine whether perinatal grief was associated with posttraumatic stress symptoms, and if so, whether grief affects posttraumatic stress symptoms through perinatal depression and anxiety (i.e., a mediation model); whether the strength of the mediated effect through perinatal depression and anxiety varies by the varying levels of social support conditions (i.e., a moderated mediation model). We put forward the following hypotheses (Fig. 1):

H1. Perinatal grief has a direct positive impact on posttraumatic stress symptoms.

H2. Perinatal grief has a direct positive impact on perinatal depression and anxiety.

H3. Perinatal depression and anxiety have a direct positive impact on posttraumatic stress symptoms.

H4. Perinatal depression and anxiety play a mediating role between perinatal grief and posttraumatic stress symptoms.

H5. Social support plays a moderating role in the relationship among perinatal grief, posttraumatic stress symptoms and perinatal depression and anxiety.

## Method

### Study design and participants characteristics

This study was conducted as a multicentre cross-sectional survey from December 2021 to October 2022 in two public hospitals in Fujian, China. It followed the guidelines of Strengthening the Reporting of Observational Studies in Epidemiology [35] (Appendix 1). The inclusion criteria were as follows: (a) women aged >18 years; (b) women hospitalized for perinatal loss; (c) women who provided informed consent and voluntary participation. The exclusion criteria included: (a) women with severe physical diseases such as heart, brain, or kidney conditions; (b) women who were unable to understand and complete the questionnaire (e.g., due to language barriers or cognitive impairments); (c) women with severe psychiatric disorders.

The sample size was calculated by PASS 15. According to preliminary experimental results, the standard deviation of the Impact of Event Scale-Revised score among 100 women with previous perinatal loss was 12.24. Given this criterion (Confidence Interval = 0.90, Confidence interval precision = 10% of the standard deviation), 269 women with previous perinatal loss were needed. After a non-response rate of 10% was considered, the minimum sample size was 296 women with previous perinatal loss.

### Measures

The questionnaires were self-reported with five categories of questions, including sociodemographic questionnaire designed by the researchers, Impact of Event Scale-Revised (IES-R), Perinatal Grief Scale (PGS), Hospital Anxiety and Depression Scale (HADS) and Multidimensional Perceived Social Support Scale (MPSS).

#### Sociodemographic questionnaire

The sociodemographic questionnaire encompassed various variables, including age, nationality, educational attainment, religious beliefs, per capita monthly household income, history of childbearing, pregnancy planning, duration of pregnancy preparation, pregnancy mode, gestational week, history of previous perinatal loss, initial perception of foetal movement, type and method of perinatal loss, and the use of analgesics during perinatal loss.

#### Impact of event scale-revised (IES-R)

The IES-R was developed by Weiss and Marmer [36] and translated by Guo et al. as the Chinese version [37]. which was a self-report measure of current subjective distress in response to a specific traumatic event. It included 22 items and each item was answered using 5-point Likert scale and the scores 0 ~ 4 were considered for not at all, a little bit, moderately, quite a bit, extremely respectively. The total score of the questionnaire was 0 ~ 88 scores. The higher the scores, the more responsive it was. The Chinese version of the IES-R was used widely and had good reliability with Cronbach’s alpha value was 0.89 and split-half reliability was 0.93 [37]. In this study, the Cronbach’s alpha value for this questionnaire was 0.91.

#### Perinatal grief scale (PGS)

The PGS was developed by Potvin [38] and translated by Xu et al. [39] as the Chinese version, which was used to measure grief in perinatal loss. It included 3 subscales with a total of 33 items: active grief, difficulty coping and despair. The items were answered using 5-point Likert scale and the scores 1 ~ 5 were considered for options of strongly disagree, disagree, neither disagree nor agree, agree, and strongly agree, respectively, and items 11 and 32 were scored reversely. The total score of the questionnaire was 33 ~ 165 scores and a score higher than 91 indicates severe grief. The Chinese version of the PGS had high internal consistency was reported for the scale with an alpha coefficient of 0.90 [39]. In this study, the Cronbach’s alpha value for this questionnaire was 0.95.

#### Hospital anxiety and depression scale (HADS)

The HADS was developed by Zigmond [40], which included 2 subscales with a total of 14 items: anxiety and depression. It was scored on a 4-point scale, and the scores 0 ~ 3 were considered for absence of the symptom, mild symptoms, moderate symptoms, severe symptoms, respectively. The total score the questionnaire was 0 ~ 42 scores. The higher the scores, the greater the heightened sense of anxiety and depression. The HADS was used widely in pregnancy loss and had good reliability with Cronbach’s alpha value ranging from 0.68 to 0.86 [7, 41]. In this study, the Cronbach’s alpha value for this questionnaire was 0.85.

#### Multidimensional perceived social support scale (MPSS)

The MPSS was developed by Zimet [42] and translated by Jiang et al. as the Chinese version [43], which included 3 subscales with a total of 12 items. It was used to measure the extent to which an individual perceives social support from three sources: Significant Others support, Family support and Friends support. It was scored a 7-point scale with ranging from ‘very strongly disagree’ (1) to ‘very strongly agree’ (7). The total score of the questionnaire was 12 ~ 84 scores. The higher the scores, the greater the heightened sense of perceives social support. The MPSS has proven to be psychometrically sound in Chinese version and to have good internal reliability and test-retest reliability, and robust factorial validity [43]. In this study, the Cronbach’s alpha value for this questionnaire was 0.95.

### Ethical approval

The study received ethical approval from the Ethical Committee of the research hospital (No: 2021YJ061), and informed consent was obtained from all participants. Since asking participants to reflect on their feelings could potentially cause distress, safeguarding procedures were implemented. Participants were provided with information about support services at the beginning and end of the survey. Furthermore, if a participant’s responses indicated particularly high posttraumatic stress symptoms, we provided details of relevant mental health helplines and advised them to contact their healthcare professional.

### Data collection

**Fig. 2:**
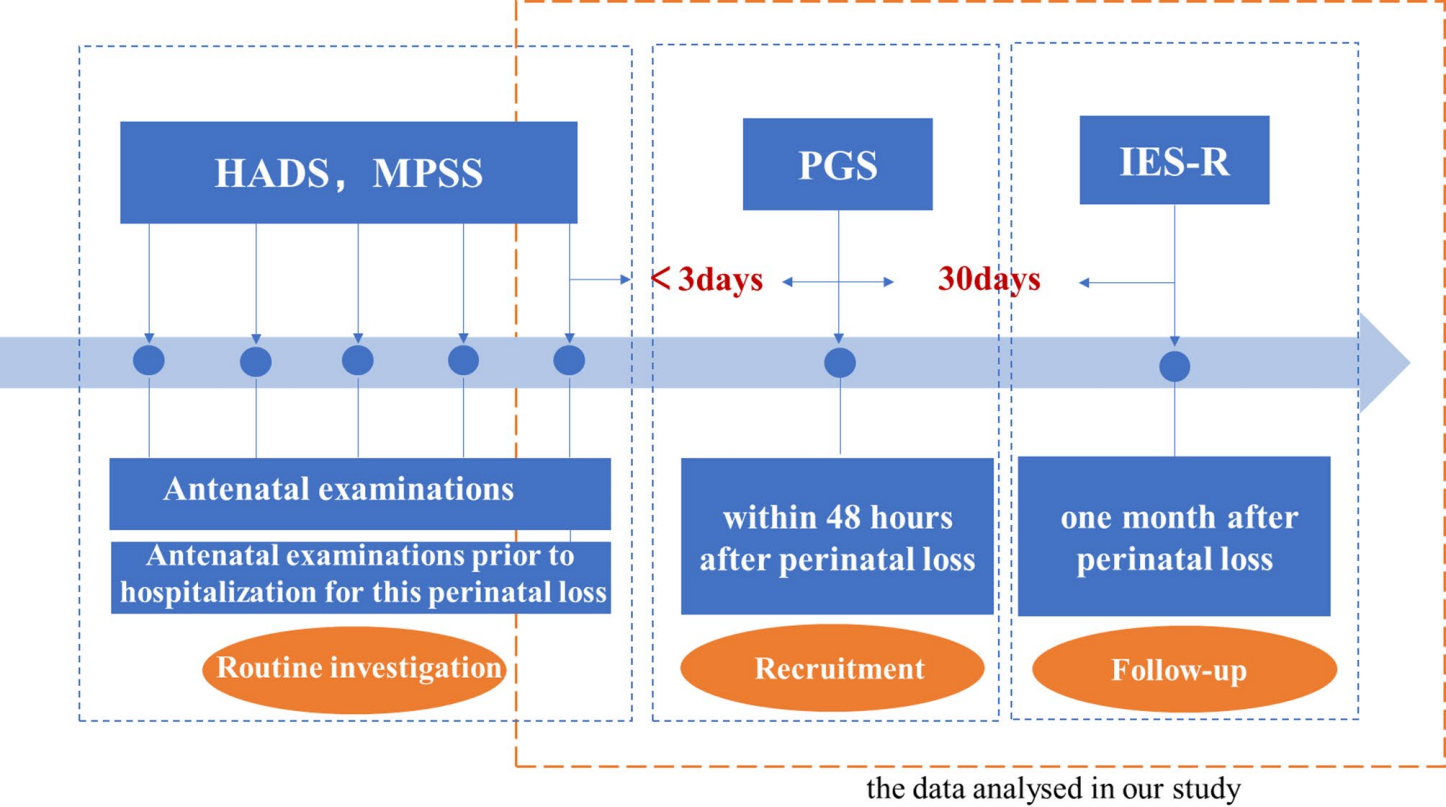
Details of the data collecting process

Data were collected across three settings: the obstetric clinic, hospital wards, and the WeChat application. To enhance service quality for prenatal care, the study hospital implemented standardized protocols for systematic evaluation of maternal psychosocial status, including routine assessments of: (a) multidimensional perceived social support (MPSS) and (b) perinatal anxiety and depression symptoms (HADS) during each antenatal examination. The study recruited participants during hospitalization for perinatal loss. In accordance with the predetermined inclusion/exclusion criteria, researchers screened potential participants for eligibility. Following informed consent procedures, the research team extracted the most recent pre-loss psychosocial assessments (MPSS and HADS scores) from obstetric clinic records as baseline measures (Antenatal examinations prior to hospitalization for this perinatal loss); administered the Perinatal Grief Scale (PGS) to participants within 48 h perinatal loss in the ward setting; and distributed the Impact of Event Scale-Revised (IES-R) through the WeChat platform at the one-month follow-up. Figure 2. presents the details of the data collecting process.

### Data analysis

The data were analysed using the SPSS (version 26.0) and the PROCESS macro. First, descriptive statistical methods (frequency, mean, standard deviation, etc.) were used. Second, we adopted Pearson’s correlation analysis to examine the correlation among four variables, perinatal grief, perinatal anxiety and depression, social support, and posttraumatic stress symptoms. There was acceptable multicollinearity, with variance inflation factor values less than 2 among independent, mediate, and moderate variables. We employed PROCESS Model 4 [44] to test the mediating role of perinatal anxiety and depression between perinatal grief and posttraumatic stress symptoms. Subsequently, Model 14 [44] was utilized to confirm the moderating role of social support in the mediating mechanism. To further understand the moderating effect of social support, a simple slopes analysis was conducted with focal points at the mean, one standard deviation above, and one standard deviation below the mean [45]. To mitigate possible alpha error inflation due to multiple testing, we computed a 95% confidence interval (95% CI) using bias-corrected percentile bootstrapping with 5000 bootstrap samples to test the effect of the independent variable on the dependent variable mediated by the mediating variable [46]. All tests were two-sided, and p-values less than 0.05 were considered statistically significant.

## Findings or results

### Participant characteristics

346 women with previous perinatal loss were selected through convenient sampling from two hospitals. Among them, 309 were valid (89.30%). The reason for the high non-response rate was that to analyse the data accurately, some data, such as records with a straight line and incomplete data were excluded in the final analysis (Appendix 2). The women with previous perinatal loss’ mean age were 30.57 years (Standard Deviations = 4.40). 91.60% participants came from ethnic Han, 65% participants had not religious beliefs, most participants had a bachelor’s degree (28.50%). Their per capita monthly household income ranged from 5000RMB to 10000RMB (1RMB = 0.1412USD), and 54.40% of the women were primipara. More than half of loss women planned a pregnancy. Other demographic data were presented in Table 1.

**Table 1:** The associations between demographic and loss-related variables and IES-R scores (*N* = 309)

Variables	Categories	N	%	IES-*R*	F	*P*
Age(years)	20–25	35	11.33%	23.40 ± 12.66	0.515	0.725
	26–30	127	41.10%	21.43 ± 12.75		
	31–35	103	33.33%	20.38 ± 12.26		
	36–40	40	12.95%	20.75 ± 12.51		
	≥ 41	4	1.29%	17.00 ± 11.75		
Nationality	ethnic Han	297	96.12%	21.15 ± 12.51	0.005	0.942
	Other	12	3.88%	21.42 ± 12.75		
Educational attainment	Junior high school and below	63	20.39%	23.00 ± 12.42	0.668	0.615
	High school and technical secondary school degree	69	22.33%	19.58 ± 12.89		
	Junior college degree	79	25.57%	21.58 ± 12.29		
	Bachelor degree	88	28.48%	20.80 ± 12.61		
	Master degree or above	10	3.24%	20.30 ± 11.52		
Did you have any religious beliefs?	Yes	108	34.95%	20.44 ± 11.75	0.556	0.457
	No	201	65.05%	21.55 ± 12.89		
Per capita monthly household income	<5000	59	19.09%	22.85 ± 13.69	0.726	0.537
	5000≤ ¥ <10,000	141	45.63%	20.12 ± 11.69		
	10,000≤ ¥ <15,000	63	20.39%	21.52 ± 11.93		
	≥ 15,000	46	14.89%	21.67 ± 14.09		
History of childbearing	multipara	141	45.63%	19.50 ± 11.90	4.642	0.032
	primipara	168	54.37%	22.55 ± 12.85		
Pregnancy planning	Yes	159	51.46%	22.10 ± 12.97	1.867	0.173
	No	150	48.54%	20.16 ± 11.94		
Pregnancy preparation time	No	151	48.87%	20.19 ± 11.90	2.422	0.066
	Within one year	137	44.34%	22.21 ± 12.80		
	One to two years	7	2.27%	12.43 ± 8.48		
	More than two years	14	4.53%	25.71 ± 15.21		
Pregnancy mode	Spontaneous conception	265	85.76%	20.64 ± 12.15	3.257	0.072
	Conception after assisted reproduction	44	14.24%	24.30 ± 14.18		
Gestational week (weeks)	≤ 12w	117	37.86%	17.95 ± 12.21	6.543	0.002
	13-27w	166	53.72%	22.95 ± 12.22		
	≥ 28w	26	8.41%	24.15 ± 12.93		
History of previous perinatal loss	Yes	144	46.60%	21.76 ± 12.90	0.632	0.427
	No	165	53.40%	20.63 ± 12.15		
Initial perception of fetal movement	Yes	116	37.54%	23.54 ± 12.07	6.892	0.009
	No	193	62.46%	19.73 ± 12.56		
Type of perinatal loss	Ectopic gestation	84	27.18%	18.25 ± 13.07	3.821	0.01
	Fetal malformation	106	34.30%	21.67 ± 11.98		
	Spontaneous abortion	45	14.56%	25.87 ± 13.05		
	Missed abortion	74	23.95%	20.86 ± 11.48		
Mode of perinatal loss	Curettage without pain	8	2.59%	16.13 ± 13.20	2.792	0.018
	Spontaneous abortion	27	8.74%	25.07 ± 14.32		
	Medical abortion (conservative treatment)	90	29.13%	18.36 ± 11.36		
	Medical abortion and painless curettage	15	4.85%	23.27 ± 11.95		
	Drug abortion and have pain curettage	104	33.66%	23.55 ± 11.77		
	Operative treatment	65	21.04%	19.72 ± 13.51		
Were analgesics used during perinatal loss	Yes	117	37.86%	20.77 ± 13.15	0.182	0.67
	No	192	62.14%	21.40 ± 12.11		

### Descriptive statistics and the correlation analyses

The mean, standard deviations and correlation coefficient results of these variables were presented in Table 2. The average score of posttraumatic stress symptoms was (21.16 ± 12.50), perinatal grief was (76.47 ± 18.62), perinatal anxiety and depression was (16.82 ± 9.39), and social support was (68.23 ± 11.07).

**Table 2:** Descriptive statistics and bivariate correlations between study variables (*N* = 309)

	M	SD	Depression and anxiety	depression	anxiety	Social support	Significant other support	Family support	Friend support	Perinatal grief	active grief	difficulty coping	despair	Posttraumatic stress symptoms	Intrusion	hyperarousal	avoidance
Depression and anxiety	16.82	9.39	1														
depression	10.75	6.28	0.986^**^	1													
anxiety	6.07	3.37	0.952^**^	0.888^**^	1												
Social Support	68.23	11.07	− 0.399^**^	− 0.425^**^	− 0.321^**^	1											
Significant other support	22.22	4.20	− 0.372^**^	− 0.402^**^	− 0.290^**^	0.927^**^	1										
Family support	23.72	3.69	− 0.284^**^	− 0.299^**^	− 0.236^**^	0.845^**^	0.683^**^	1									
Friend support	22.28	4.44	− 0.406^**^	− 0.431^**^	− 0.330^**^	0.914^**^	0.797^**^	0.630^**^	1								
Perinatal grief	76.47	18.62	0.636^**^	0.632^**^	0.596^**^	− 0.429^**^	− 0.386^**^	− 0.355^**^	− 0.408^**^	1							
active grief	30.22	7.76	0.570^**^	0.559^**^	0.549^**^	− 0.293^**^	− 0.278^**^	− 0.230^**^	− 0.277^**^	0.897^**^	1						
difficulty coping	23.01	6.06	0.584^**^	0.586^**^	0.538^**^	− 0.469^**^	− 0.410^**^	− 0.406^**^	− 0.444^**^	0.884^**^	0.628^**^	1					
despair	23.25	6.62	0.585^**^	0.586^**^	0.540^**^	− 0.432^**^	− 0.385^**^	− 0.357^**^	− 0.416^**^	0.950^**^	0.776^**^	0.833^**^	1				
Posttraumatic stress symptoms	21.16	12.50	0.447^**^	0.452^**^	0.404^**^	− 0.241^**^	− 0.218^**^	− 0.217^**^	− 0.214^**^	0.488^**^	0.459^**^	0.432^**^	0.439^**^	1			
Intrusion	7.35	4.20	0.362^**^	0.368^**^	0.324^**^	− 0.200^**^	− 0.169^**^	− 0.170^**^	− 0.198^**^	0.444^**^	0.413^**^	0.385^**^	0.410^**^	0.899^**^	1		
hyperarousal	4.61	4.27	0.456^**^	0.461^**^	0.415^**^	− 0.240^**^	− 0.231^**^	− 0.208^**^	− 0.208^**^	0.463^**^	0.420^**^	0.427^**^	0.420^**^	0.810^**^	0.639^**^	1	
avoidance	9.20	5.85	0.362^**^	0.366^**^	0.328^**^	− 0.196^**^	− 0.176^**^	− 0.190^**^	− 0.163^**^	0.387^**^	0.379^**^	0.335^**^	0.337^**^	0.901^**^	0.737^**^	0.544^**^	1

Perinatal depression and anxiety were measure by Hospital Anxiety and Depression Scale (HADS); Perinatal Grief were measure by Perinatal Grief Scale (PGS); Social support were measure by Multidimensional Perceived Social Support Scale (MPSS); Posttraumatic stress symptoms were measure by Impact of Event Scale-Revised (IES-R); Note: *P<.05, **P<.01

Pearson’s correlation analysis showed that perinatal grief was positively correlated with posttraumatic stress symptoms (*r* =.488, *P* <.01) and perinatal anxiety and depression (*r* =.636, *P* <.01). Perinatal anxiety and depression were positively correlated with posttraumatic stress symptoms (*r =*.447, *P <*.01), social support was negatively correlated with the perinatal anxiety and depression (*r* = −.399, *P* <.01), perinatal grief (*r* = −.429, *P* <.01), (H1, H2, H3 was supported).

### Moderated mediation models

**Fig. 3:**
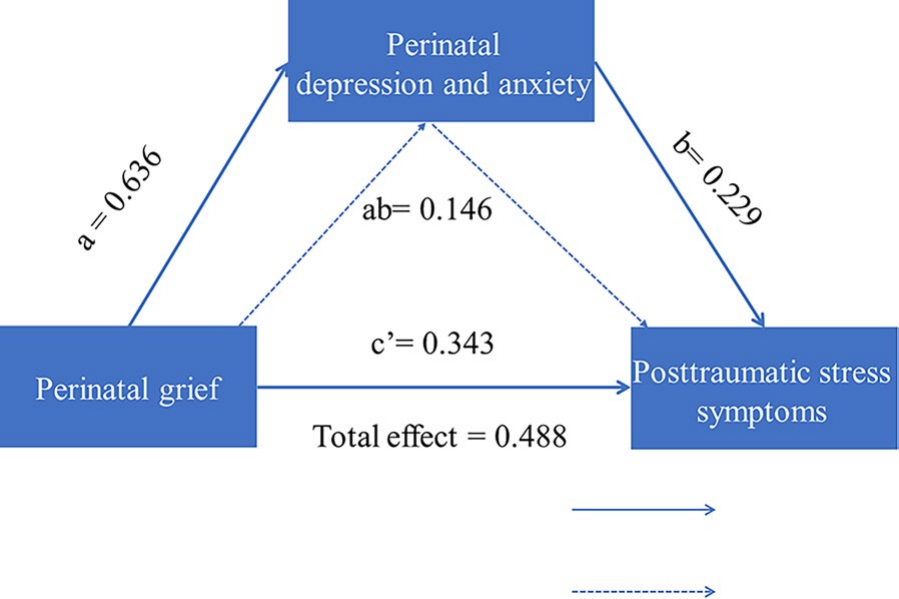
Mediating effect of perinatal depression and anxiety symptoms

Firstly, SPSS PROCESS compiled by Hayes [44] were adopted Model 4 (a simple mediation model) tested the mediating effect of perinatal anxiety and depression on the relationship between the perinatal grief and posttraumatic stress symptoms. The results showed that the perinatal grief positively predicted posttraumatic stress symptoms (*β* = 0.488, *t* = 9.802, *P*<.001). When the perinatal anxiety and depression and perinatal grief entered regression equation together, and the perinatal grief could predict posttraumatic stress symptoms (*β* = 0.343, *t* = 5.412 *P* <.001). Perinatal grief also could positively predict perinatal depression and anxiety (*β* = 0.636, *t* = 14.438, *P* <.001), and perinatal anxiety and depression could positively predict posttraumatic stress symptoms (*β* = 0.229, *t* = 3.617, *P* <.001). All data were presented in Table 3. The PROCESS bootstrapping procedures were used to test the significance of the indirect effects, and results could be seen in Fig. 3; Table 4. The indirect effect of perinatal grief through perinatal anxiety and depression on posttraumatic stress symptoms was significant (*β* = 0.146, *SE* = 0.044, 95%*CI*= [0.062, 0.235]). In addition, *R*^*2*^ values showed that including the two mediators into the model more than the variance explained by the model (*R*^*2*^ mediation model = 0.270; *R*^*2*^ total effect model = 0.238). The proportion of indirect effect and direct effect to total effect was 29.80%, 70.20% respectively (H4 was supported).

**Table 3:** Indirect effect of perinatal grief on posttraumatic stress symptoms mediated by perinatal depression and anxiety symptoms (Model 4)

	Z Depression and anxiety symptoms			Z Posttraumatic stress symptoms			Z Posttraumatic stress symptoms		
	β	SE	t	β	SE	t	β	SE	t
Z Perinatal grief	0.636	0.440	14.438^**^	0.343	0.063	5.412^**^	0.488	0.498	9.802^**^
Z Depression and anxiety symptoms	/	/	/	0.229	0.063	3.617^**^	/	/	/
*R*	0.636			0.519			0.488		
*R-sq*	0.404			0.270			0.238		
*F*	208.447			56.472			96.082		

SE, Standard error

*P<.05,**P<.01

**Table 4:** Total effect、direct effect and indirect effect of perinatal grief on posttraumatic stress symptoms

Estimate	β	Boot SE	Boot LLCI	Boot ULCI	Effect ratio
**Indirect effect**					
perinatal grief → posttraumatic stress symptoms	0.146	0.044	0.062	0.235	0.298
**Direct effect**					
perinatal grief → posttraumatic stress symptoms	0.343	0.062	0.220	0.463	0.702
**Total effect**					
perinatal grief → posttraumatic stress symptoms	0.488	0.050	0.389	0.586	

Boot SE, Standard Error of indirect effects estimated by Bootstrap method test with percentile bias; Boot LLCI, 95% confidence interval lower; Boot ULCI, 95% confidence interval upper

Second, the Model 14 of SPSS PROCESS by Hayes [44] was utilized to test the moderating effect of social support in the mediating mechanism. Moderated mediation results were shown in Table 5. The interaction between perinatal anxiety and depression, and social support on posttraumatic stress symptoms demonstrated a significant effect (*β* = −0.103, *t* = 0.044, *P* <.01), In addition, *R*^*2*^ values in the moderating model was 0.282. This finding suggested that social support moderated the relationship among perinatal grief, posttraumatic stress symptoms and perinatal depression and anxiety (H5 was supported).

**Table 5:** Indirect effect of perinatal grief on posttraumatic stress symptoms mediated by perinatal depression and anxiety symptoms and moderated by social support conditions (Model 14)

	Z Posttraumatic stress symptoms			Z Depression and anxiety symptoms		
	β	SE	t	β	SE	t
constant	−0.041	0.052	−0.791	0.000	0.044	0.000
Z Perinatal grief	0.360	0.066	5.504^**^	0.636	0.044	14.438^**^
Z Depression and anxiety symptoms	0.208	0.065	3.214^**^	/	/	/
Z Social support	0.028	0.056	0.493	/	/	/
Z Depression and anxiety symptoms *Z social support	−0.103	0.044	−2.316^*^	/	/	/
*R*	0.531			0.636		
*R-sq*	0.282			0.404		
*F*	29.888			208.447		

SE, Standard Error

*P<.05,**P<.01

**Fig. 4:**
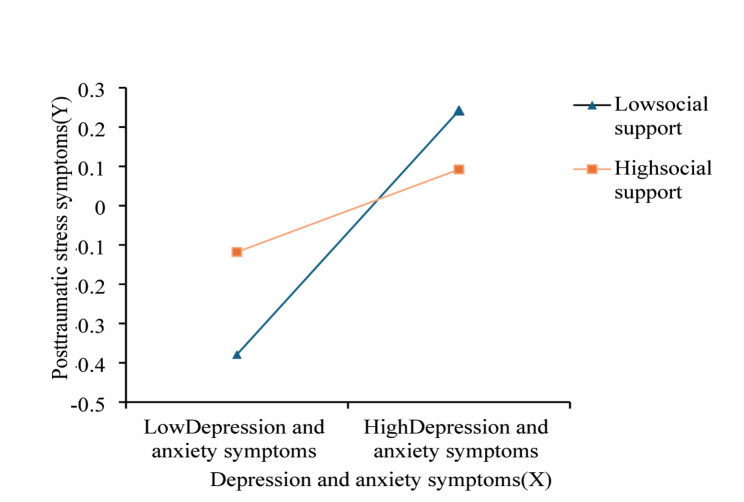
The moderating effect of social support conditions on the relationship between perinatal depression and anxiety symptoms and posttraumatic stress symptoms

Additionally, the simple slope analysis results showed that the effect of perinatal anxiety and depression on posttraumatic stress symptoms decreased as social support increased (Fig. 4; Table 6). There was an increasing trend in coefficients for social support one standard deviation below the mean (β = 0.310, 95% CI = 0.166–0.454), at the mean (β = 0.208, 95% CI = 0.081–0.335), and one standard deviation above the mean (β = 0.105, 95% CI = −0.059-0.269). With higher social support, there was a weaker positive relationship between perinatal anxiety and depression, and posttraumatic stress symptoms, compared to situations with lower social support.

**Table 6:** Direct effects of perinatal anxiety and depression symptoms on posttraumatic stress symptoms moderated by different levels of social support

	Z Social support	*β*	Boot SE	Boot LLCI	Boot ULCI
moderated mediation model	Eff1(M-1SD)	0.310	0.073	0.166	0.454
	Eff2(M)	0.208	0.065	0.081	0.335
	Eff3(M + 1SD)	0.105	0.083	−0.059	0.269

Z Social Support, Standardized Social Support were measure by Multidimensional Perceived Social Support Scale; Boot SE, Standard Error of indirect effects estimated by Bootstrap method test with percentile bias; Boot LLCI, 95% confidence interval lower; Boot ULCI, 95% confidence interval upper

## Discussion

This study aimed to investigate the relationship among women with previous perinatal loss’s perinatal grief, perinatal anxiety and depression, social support and posttraumatic stress symptoms. The findings indicated that perinatal grief, perinatal anxiety and depression as well as social support, were all correlated with posttraumatic stress symptoms, however, the effects of these factors were different. Meanwhile, the present study confirmed that perinatal grief had a direct effect on posttraumatic stress symptoms, with perinatal anxiety and depression playing a mediating role. We also found that social support played a moderating role in the relationship among perinatal grief, perinatal anxiety and depression, and posttraumatic stress symptoms. Specifically, perinatal anxiety and depression had a stronger predictive effect on posttraumatic stress symptoms in women with low social support, as compared to those with high social support.

The result showed that perinatal grief positive effect posttraumatic stress symptoms in women who have experienced perinatal loss. These results were consistent with Davoudian’s study, highly intense perinatal grief had been linked to the development of posttraumatic stress symptoms [47]. Approximately 25%−30% of women who have experienced perinatal loss may experience significant, prolonged, and intensely complicated grief reactions, which can detrimentally affect their long-term psychological well-being [16]. A meta-analysis revealed that peritraumatic emotional responses were the second strongest predictor of posttraumatic stress symptoms [48]. Women who exhibit elevated negative peritraumatic emotional responses, including grief, intense fear, helplessness, and loss of control, may enter a dissociative state as a coping mechanism to distance themselves from the emotional event [49]. This peritraumatic dissociation has been linked to a heightened sense of perceived threat and an external locus of control [50]. As a result, the persistence of trauma may occur, subsequently leading to the development of posttraumatic stress symptoms.

This study demonstrates a significant positive correlation between perinatal anxiety and depression and posttraumatic stress symptoms, consistent with prior findings in stillbirth-related research [51]. Furthermore, existing evidence suggests that women with compromised psychological well-being during pregnancy exhibit heightened vulnerability to traumatic experiences, thereby increasing the likelihood of posttraumatic stress symptoms development [52]. The higher the level of perinatal anxiety and depression in women, the greater the risk of developing posttraumatic stress symptoms after perinatal loss. There are several possible reasons to explain it. First, individuals with high levels of depression often exhibit an amplified interpretation of negative information and an overestimation of personal risk [53]. Therefore, women with heightened levels of anxiety and depression were more inclined to interpret perinatal loss in a manner that fosters the emergence of posttraumatic stress symptoms. Second, perinatal anxiety and depression could adversely affect a mother’s sense of control during childbirth, thereby increasing the risk of developing posttraumatic stress symptoms [54]. Meanwhile, anxiety, depression, and posttraumatic stress symptoms can all stem from neuroendocrine disorders in individuals, and their interactive effects may elucidate their interconnectedness [55].

This study also revealed an association between perinatal grief and perinatal anxiety and depression, which in turn, were positively correlated with posttraumatic stress symptoms. These findings are consistent with the research conducted by Gaudet [34] and Lindström [56]. Posttraumatic stress symptoms were distinctive psychological state that emerges following the experience of a traumatic event [20], which was a consequence of the intricate interplay among multiple factors, encompassing pre-traumatic mental state, the traumatic event, and other relevant variables [20]. A retrospective study showed that women with previous perinatal loss with higher levels of perinatal grief have experienced high anxiety and depression symptoms during pregnancy [56]. Additionally, women with high perinatal anxiety and depression are more susceptible to the impact of significant events, and then may subsequently develop post-traumatic stress disorder [57]. The results of this study further confirm this view, posttraumatic stress symptoms of women with previous perinatal loss were influenced by multiple factors like perinatal grief, perinatal anxiety and depression and so on. Additionally, perinatal grief serves as a predictor of posttraumatic stress symptoms, mediated by perinatal anxiety and depression. Therefore, beyond simply reducing perinatal grief, attention should also be paid to perinatal anxiety/depression. Perinatal depression/anxiety-based psychotherapeutic interventions may help reduce posttraumatic stress symptoms among women experiencing perinatal loss [58]. Bereavement counselling has also been found effective in reducing grief in the long run [59]. Another study found that online perinatal loss groups can reduce grief among women who have experienced perinatal loss. In a virtual community space, women with similar experiences of perinatal loss can communicate with each other and express their feelings within the group [60]. Therefore, future research could further explore whether combining face-to-face bereavement counselling with online perinatal loss groups is more effective in alleviating grief among women who have experienced perinatal loss.

The research findings demonstrated the significant role of social support as a moderator in the relationship among perinatal grief, perinatal anxiety and depression and posttraumatic stress symptoms. Women with previous perinatal loss with high social support exhibited a diminished direct predictive effect of perinatal anxiety and depression on posttraumatic stress symptoms, in contrast to those with low social support. This indicate that social support as a protective factor can alleviate posttraumatic stress symptoms resulting from various factors. These findings were consistent with prior research, underscoring that women who receive substantial social support exhibit a diminished risk of developing posttraumatic stress symptoms subsequent to perinatal loss [32]. Individuals with high levels of social support typically demonstrated superior emotional regulation abilities and exhibited composed, logical, and proactive responses when confronted with trauma. These qualities consequently diminish the risk of developing posttraumatic stress symptoms [61, 62]. Moreover, individuals with heightened social support tended to actively seek emotional and practical assistance from others, facilitating a re-evaluation of the event with reduced negative emotional bias. This, in turn, results in a decrease in negative emotions associated with the event [63]. Hence, sufficient social support could aid in alleviating pregnancy-related anxiety and depression among women who have experienced perinatal loss, thereby reducing the probability of developing posttraumatic stress symptoms. Listening and sympathizing are important ways of providing social support to alleviate negative emotions among these women [64]. They expressed their need to talk about their experience and to have someone listen, finding it most helpful to talk with someone who truly understood what they were going through [64]. Therefore, midwives should listen more and encourage caregivers, family, and friends to listen and provide emotional support. Additionally, midwives can establish women with previous perinatal loss’s club [65], which provides a platform for sharing and communication for all women who have experienced perinatal loss.

This study was conducted from December 2021 to October 2022, during the novel coronavirus epidemic. At that time, China’s general COVID-19 prevention and control policy was the “Dynamic COVID-Zero Strategy” [66]. Fuzhou, where the survey was conducted, was classified as a low-risk area with only single-digit or even zero positive infections. Pregnant women’s travel, medical treatment, and social interactions were normal. Additionally, our survey involved filling out a questionnaire (with face-to-face interaction lasting approximately 5 min for obtaining informed consent and providing instructions for filling it out), as well as online follow-up on the WeChat app. Given these circumstances, the recruitment process and social support among perinatal loss individuals may not have been affected by COVID-19 restrictions. Moreover, all items on the perinatal grief scale we used focused on pregnancy-related loss, and the women who experienced perinatal loss in this study had not reported any other recent traumatic adverse events. Therefore, the grief among individuals experiencing perinatal loss may not have been affected by COVID-19 restrictions. Pregnant women are susceptible to anxiety and depression due to hormonal fluctuations, alterations in body shape, role transitions, and concerns regarding fatal health [21]. Approximately 16% of women experience depression and anxiety during pregnancy [67]. During the COVID-19 pandemic, symptoms of anxiety and depression are more pronounced in pregnant women [68]. We cannot rule out the effect of COVID-19 on the negative emotions of women experiencing perinatal loss in this study. Therefore, future studies need to more fully consider the social environment of the study subjects.

The study had several limitations that should be acknowledged. Firstly, our study verifies the hypothesized mediation model based on cross-sectional data. However, the cross-sectional design limits causal inferences. Thus, future research should adopt more rigorous experimental designs and analytical methods (multi-wave longitudinal designs, cross-lagged panel models, or intervention experiments) to validate the mediation mechanisms. For instance, a prospective cohort study could be conducted by recruiting pregnant women with and without anxiety and depression following perinatal loss, followed by longitudinal assessments at 3 months, 6 months, and 1 year after perinatal loss. This approach would help determine the incidence of post-traumatic stress symptoms and clarify whether antenatal anxiety and depression contribute to the development of post-traumatic stress disorder (PTSD). Secondly, due to methodological limitations, this study could not reliably assess the specific impact of COVID-19 containment strategies on the severity of prenatal anxiety and depressive. Finally, this study employed self-report questionnaires for data collection. While this approach facilitates efficient screening of participants’ positive outcomes, it is susceptible to biases such as recall bias and social desirability bias. These limitations may compromise the validity of assessments related to prenatal anxiety and depression, perceived social support, and post-traumatic stress disorder, thereby confounding the interpretation of intervariable relationships. Future studies should adopt multimodal assessments-for instance, integrating objective biomarkers (e.g., cortisol levels) and collateral reports from healthcare providers or family members-to enable methodological triangulation and mitigate the influence of self-report biases.

## Conclusion

Overall, the findings have important implications for interventions that seek to reduce posttraumatic stress symptoms in women with previous perinatal loss with high levels of grief. It was observed that the grief after perinatal loss positively affected posttraumatic stress symptoms, perinatal anxiety and depression positively affected posttraumatic stress symptoms, and perinatal anxiety and depression mediated between perinatal grief and posttraumatic stress symptoms. In addition, anxiety and depression of women with previous perinatal loss with high social support has a weaker predictive effect on posttraumatic stress symptoms compared to those with low social support. Therefore, when midwives evaluate the posttraumatic stress symptoms of women with previous perinatal loss, perinatal grief, perinatal anxiety and depression should be considered together. Healthcare institutions can implement various interventions to enhance the mental health of women with previous perinatal loss, including establishing pregnant women support communities, offering mindfulness, relaxation courses [69], and utilizing structured programs (e.g., cognitive behaviour therapy) yielded sustained economic empowerment for mothers and to alleviate anxiety and depressive symptoms while improving emotional regulation [70]. Additionally, creating peer support groups for these women can provide a safe space for communication, reduce isolation, and facilitate psychological adjustment after trauma. It is also essential to strengthen follow-up systems and offer grief counselling or trauma therapy referrals for ongoing perinatal grief support. At last, healthcare professionals should encourage family and partner involvement, thereby reinforcing the social support system and engaging them in memorial activities (e.g., tree planting, letter writing), while providing emotional support through active listening and a non-judgmental approach.

## Supplementary Information

Supplementary Material 1.

## Data Availability

The datasets used and/or analysed during the current study available from the corresponding author on reasonable request.
